# Comparison and optimization of CRISPR/dCas9/gRNA genome-labeling systems for live cell imaging

**DOI:** 10.1186/s13059-018-1413-5

**Published:** 2018-03-22

**Authors:** Yu Hong, Guangqing Lu, Jinzhi Duan, Wenjing Liu, Yu Zhang

**Affiliations:** 1 Peking University-Tsinghua University-National Institute of Biological Sciences Joint Graduate Program, School of Life Sciences, Peking University, Beijing, 100871 China; 2 Graduate School of Peking Union Medical College, Beijing, 100730 China; 3 National Institute of Biological Sciences, Beijing, 102206 China

**Keywords:** Genome labeling, CRISPR/dCas9, Bimolecular fluorescence complementation (BIFC)

## Abstract

CRISPR/dCas9 binds precisely to defined genomic sequences through targeting of guide RNA (gRNA) sequences. In vivo imaging of genomic loci can be achieved by recruiting fluorescent proteins using either dCas9 or gRNA. We thoroughly validate and compare the effectiveness and specificity of several dCas9/gRNA genome labeling systems. Surprisingly, we discover that in the gRNA-labeling strategies, accumulation of tagged gRNA transcripts leads to non-specific labeling foci. Furthermore, we develop novel bimolecular fluorescence complementation (BIFC) methods that combine the advantages of both dCas9-labeling and gRNA-labeling strategies. The BIFC-dCas9/gRNA methods demonstrate high signal-to-noise ratios and have no non-specific foci.

## Background

The dynamic localization of a particular genomic locus in a three-dimensional (3D) genome has been proposed to regulate various genome functions including gene transcription, DNA recombination, DNA replication, and DNA repair [1, 2]. Until recently, several strategies have been developed to trace the dynamic movement of genomic loci in living cells [3]. Clustered regularly interspaced short palindromic repeats (CRISPR)/CRISPR-associated protein 9 (Cas9), an RNA-guided endonuclease that mediates highly sequence-specific binding and efficient cleavage on genomic DNA, has been extensively developed recently for genome editing [4–7]. On the other hand, a nuclease-deficient Cas9 (dCas9) could bind to a guide RNA (gRNA)-specific genomic locus, where by recruiting various effectors it could achieve precise and programmed transcription activation and repression, epigenetic remodulations of local histone and DNA modifications, labeling and visualization of the genomic locus, and single base genome mutagenesis [8–10]. Various dCas9/gRNA systems have been designed to label genomic loci in live cells. In its first version, direct fusion of fluorescent proteins such as green fluorescent protein (GFP) with dCas9 protein was used by Huang’s laboratory [11]. To increase the signals, a SunTag that contains multiple copies (24X) of GCN4 peptide epitopes has been added to the C-terminal dCas9 [12]. Fusion with single-chain fragment variable (scFv) antibody against GCN4 peptide allows more copies of fluorescent proteins to be recruited to a single tethered dCas9/gRNA complex. Recently, tandem FP11-tags were also fused to dCas9 to allow proportional enhancement of the fluorescence signal [13]. To achieve simultaneous labeling of several genomic loci at the same time, two approaches have been developed. First, several CRISPR/Cas9 orthologous proteins from distinct bacterial species that have different gRNA-binding specificities could be fused to different fluorescent proteins [14, 15]. On the other hand, both RNA aptamer binding effectors [16–19] and Pumilio/FBF (PUF) RNA-binding proteins [20] have been utilized to label the different gRNAs, which could work with the same dCas9 protein. In addition, multiple copies of RNA motifs could be fused to the gRNA to greatly amplify the signals. Here we compare several of the latest gRNA labeling and dCas9 labeling systems in the same experimental settings such as the cell type, transfection method, and gRNA expression cassette, as well as genomic targets. We have identified and solved the intrinsic nonspecific labeling issue associated with the gRNA labeling methods. We also developed novel bimolecular fluorescence complementation (BIFC)-dCas9/gRNA methods that combine the specificities of both dCas9 and gRNAs. The BIFC-dCas9/gRNA methods have high signal-to-noise ratio and no nonspecific foci.

## Results

### Comparison of dCas9/gRNA genome-labeling systems for chromosome imaging

**Fig. 1:**
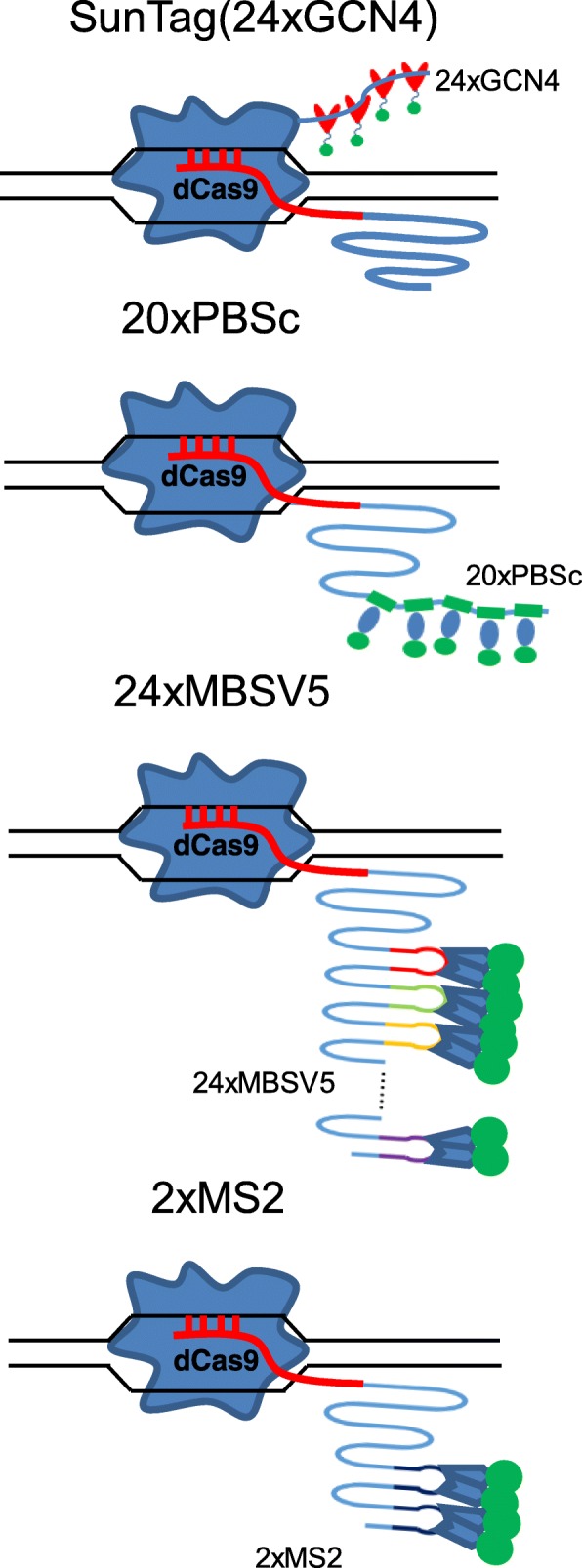
Schematic views of the different CRISPR/dCas9 genome-labeling systems. In the SunTag system, nuclease-deficient CRISPR/Cas9 (*dCas9*) protein is fused with a 24X repeating GCN4 peptide array, which could recruit multiple copies of scFv-GFP, thereby enabling labeling of specific genomic loci in living cells. The *Casilio* (20XPBSc) system consists of the dCas9 protein, a gRNA appended with 20xPUF-binding sites (gRNA-20XPBS), and fluorescent proteins fused with a PUF domain. The 2XMS2 system contains the dCas9 protein, a gRNA containing 3’ 2XMS2 hairpins that can recruit four molecules of MS2 coating protein (MCP)-GFP. 24XMBSV5 contains 24X synonymous MS2 hairpins

**Fig. 2:**
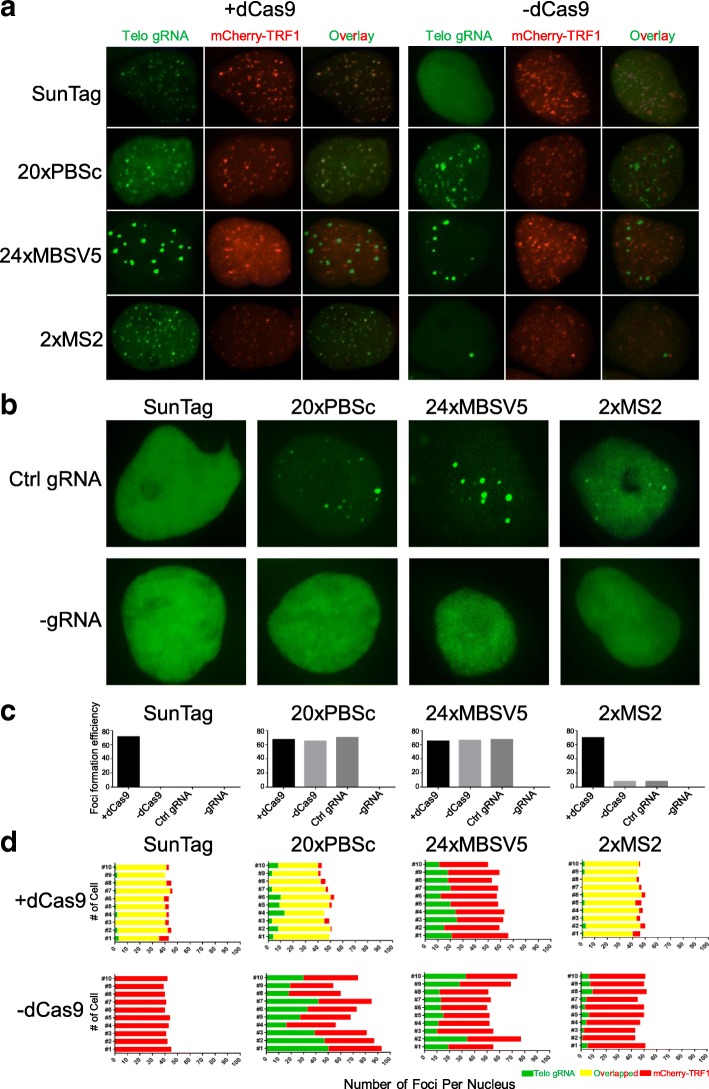
Comparison of different dCas9/gRNA systems for labeling human telomeres. **a** The dCas9-labeling (SunTag) and gRNA-labeling (20XPBSc, 24XMBSV5, and 2XMS2) systems were tested in HEK293T cells. A gRNA-targeting human telomere repeat was transfected together with dCas9 *(+dCas9*, *left panel*) or without dCas9 *(–dCas9*, *right panel*). The mCherry-TRF1 was co-transfected to label telomeres. **b** Representative images of the negative controls (with control gRNA and without gRNA) for different CRISPR/dCas9 labeling systems. **c** The percentages of cells having dCas9/gRNA foci in all GFP positive cells (*N* ≥ 30) were compared for difference labeling systems. Negative controls include without dCas9, with control gRNA, and without gRNA (–gRNA). **d** Quantification of telomere labeling specificity, in the condition with (*upper panel*) and without dCas9 (*lower panel*), based on co-localization with mCherry-TRF1 signals

We directly compared SunTag for dCas9 labeling and 20XPBSc (PUF-binding site c) (*Casilio*) [20], 24XMBSV5 (MS2-binding site v5) [21], and 2XMS2 hairpins [17] for 3’ gRNA labeling of human telomeres in human embryonic kidney 293T (HEK293T) cells (Figs. 1 and 2). The mCherry-TRF1 [22] was co-transfected to verify the specificity of dCas9/gRNA labeling. Several negative controls were also included. As shown in the first panel of Fig. 2a–d, in the SunTag system, co-transfection with gRNA-targeting telomere repeat sequences resulted in telomere foci which co-localized with mCherry-TRF1 foci in all transfected cells. Importantly, there were absolutely no nonspecific foci in the negative control samples co-transfected with the control gRNA expression vector, as well as in those transfected without dCas9 or gRNA. On the other hand, although 20XPBSc showed similar telomere-labeling foci to those of the SunTag system, in transfection without dCas9 or transfection with the same amount of control gRNA, similar percentages of cells showed significant numbers of nonspecific foci that did not co-localize with mCherry-TRF1 (Fig. 2a–d, the second panel). These data suggested that in the *Casilio* system at least some foci observed when dCas9 and telomere gRNA were transfected might be nonspecific. For gRNA 3′ labeled with 24XMBSV5, which contains 24X synonymous MS2-binding sites, surprisingly, the resulted foci could not overlap with mCherry-TRF1 at all in transfections both with and without dCas9 (Fig. 2a–d, the third panel). Interestingly, after the numbers of MS2-binding motif were reduced to 2X, the percentages of cells with nonspecific foci and the numbers of those foci in each cell were both significantly decreased when dCas9 was omitted or control gRNA was used (Fig. 2a–d, the fourth panel). When 2XMS2 was fused to stem loops in the middle of gRNA (CRISPRainbow [17]), it behaved the same as 3’ 2XMS2 (see Ref. [23], “Comparison and optimization of CRISPR/dCas9/gRNA genome labeling systems for live cell imaging Additional file 1”: Figure S1). In addition, we observed such nonspecific foci in the absence of dCas9 in other mouse and human cell types including B16, U2OS, and HeLa cells ([23] Additional file: Figures S2–S4).

**Fig. 3:**
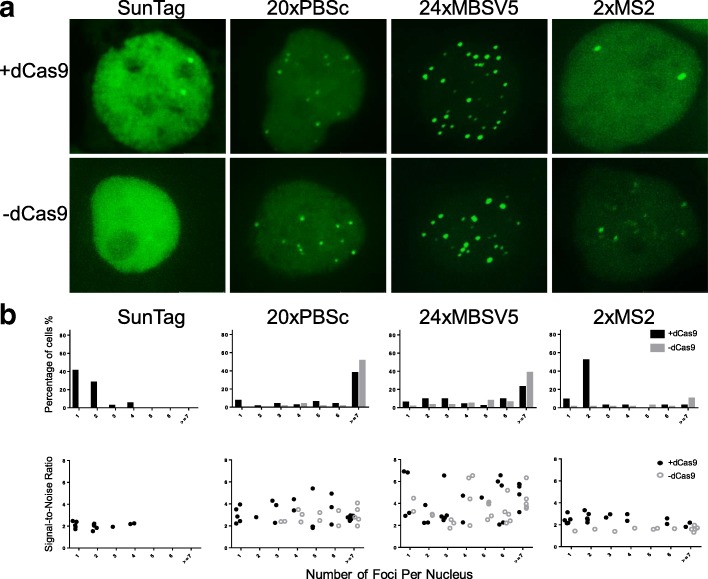
Comparison of different dCas9/gRNA systems for labeling the endogenous human MUC4 genomic locus. **a** A gRNA targeting the human *MUC4* gene was transfected together with dCas9 (*upper panel*) or without dCas9 (*lower panel*). **b** *Upper panel*: histograms of dCas9/gRNA foci formation efficiency in different dCas9/gRNA labeling systems (measured as % of GFP-positive cells, *N* > =20) in the condition with dCas9 (*black bars*) and without dCas9 (*gray bars*). *Lower panel*: dot plots of signal-to-noise ratio in the condition with dCas9 (*black dots*) and without dCas9 (*gray dots*). Each dot represents average value of all foci in one cell

We also compared these different systems for labeling a single genomic locus such as the *MUC4* gene in chromosome 3 containing 90 gRNA-targeting repeats [11]. In SunTag system-transfected cells, as expected, the cells contained mostly one or two foci when dCas9 was transfected, whereas no foci were observed in the absence of dCas9 (Fig. 3a and b, the first panel). On the contrary, in MUC4e-20XPBSc and MUC4e-24XMBSV5 gRNA-transfected cells, most of the cells contained more than six foci in each nucleus both with and without dCas9 (Fig. 3a and b, the second and third panels). After reducing MS2 hairpins to 2X, when dCas9 was co-transfected, the labeling pattern was more similar to that in the SunTag system, although there were still a few cells having more than six foci. On the other hand, in the absence of dCas9, the nonspecific foci for 3’ 2XMS2 gRNA were similar to those observed in the MUC4e-20XPBSc and MUC4e-24XMBSV5 gRNA-transfected cells. We also measured the signal-to-noise ratio (background noise was defined as fluorescence intensity of the nucleus, which most likely was generated by free unbound fluorescent proteins) for foci observed in these labeling systems. For MUC4e-20XPBSc and MUC4e-24XMBSV5, the signal-to-noise ratio showed no significant differences between samples with and without dCas9. However, the signal-to-noise ratios of the nonspecific foci observed in MUC4e-2XMS2 without dCas9 samples were lower than those of the foci with dCas9. We also tested another genomic region in chromosome 3 (around 197 Mb) containing 48 gRNA-targeting repeats and obtained similar results (see Ref. [23], Additional file: Figure S5).

### Dissection of the nonspecific labeling foci associated with gRNA labeling for chromosome imaging

**Fig. 4:**
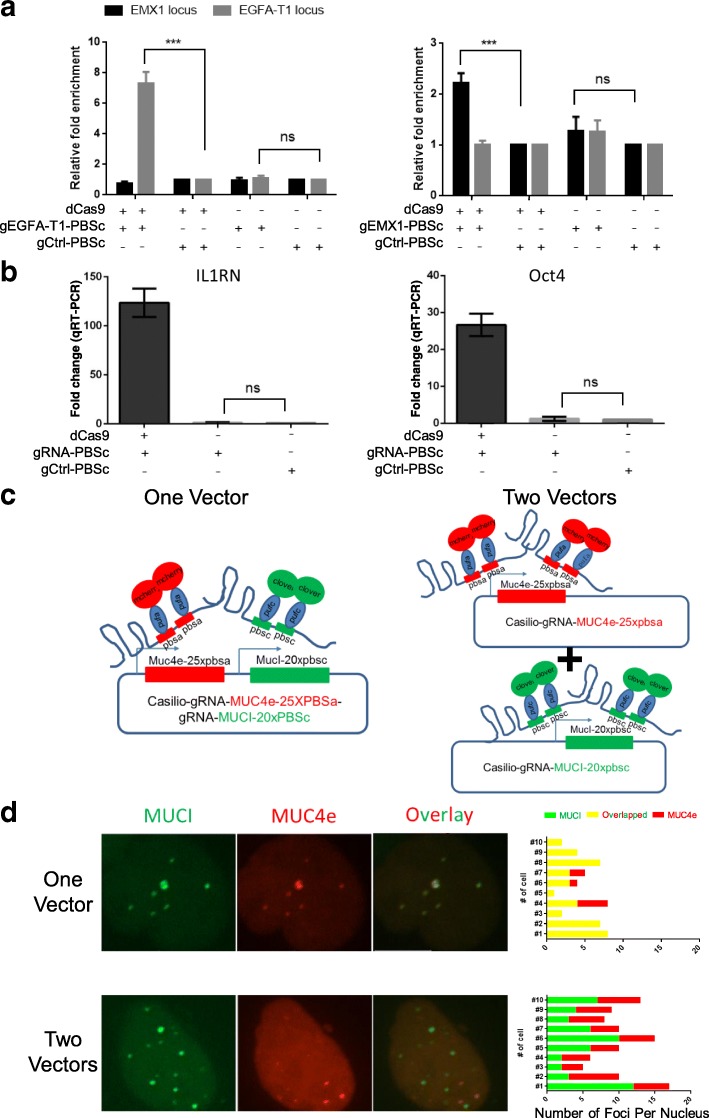
Dissection of nonspecific labeling foci associated with gRNA-labeling systems. **a** ChIP-qPCR results demonstrate that the specific binding of gRNAs to their endogenous targets is dependent on dCas9. Relative enrichment levels of GFP-tagged PUFc at the targeted loci by EGFA-T1-gRNA (*left*), EMX1-gRNA (*right*), and control loci were compared in the conditions with and without dCas9. **b** Targeted gene transcription activation by *Casilio* system is also dependent on dCas9. PUFc-VP64 and gRNA-5 × PBSc targeting IL1RN or Oct4 promoter were transfected into HEK293T cells with or without dCas9. RT-qPCR was performed to evaluate the fold changes of IL1RN and Oct4 expression. **c** Schematic of the “one vector” and “two vectors” settings to dissect the nonspecific foci observed in gRNA labeling systems. **d** Nonspecific labeling foci came from accumulation of gRNA transcripts surrounding the gRNA transcription cassettes. In “one vector,” a single plasmid containing expression cassettes for both MUCI and MUC4e gRNAs was used. In “two vectors,” two plasmids containing individual MUCI and MUC4e gRNA expression cassettes were transfected. The representative images and quantifications are shown. The data are displayed as mean ± standard deviation (SD) from at least three independent experiments. Unpaired *t* test was used. ***p < 0.001; *ns* not significant

We employed several approaches to dissect where those gRNA-dependent nonspecific foci might come from. First, chromatin immunoprecipitation (ChIP) was performed for two gRNA-targeted endogenous genomic loci, EGFA-T1 and EMX-1 [24]. As shown in Fig. 4a, transfection of dCas9 and gRNA containing 20XPBSc could significantly enrich the gRNA-specific binding of PUFc-Clover on the targeted genomic region. However, such enrichments were completely abolished when dCas9 was not co-transfected, suggesting that the nonspecific foci are not the on-target sequences. We also employed a more sensitive CRISPR/dCas9 transcription activation system [20] to confirm these results (Fig. 4b). Endogenous IL1RN and Oct4 promoters could be efficiently activated by dCas9, gRNA-20XPBSc targeting respective promoters, and PUFc-VP64. Similar to the ChIP results, omitting dCas9 completely abolished such activation. Finally, to test the hypothesis that such nonspecific foci might originate from gRNA transcripts that are closely tethered to transfected gRNA-expressing plasmids, we transfected HEK293T cells with a single vector containing both expression cassettes for MUC4e-25XPBSa and MUCI-20XPBSc gRNAs (one vector) or two individual vectors containing those two gRNA expression cassettes separately (two vectors) (Fig. 4c). Indeed, in the absence of dCas9 expression, the MUC4e (red) and MUCI (green) nonspecific foci in the “one vector” transfection could mostly be overlapped, whereas they were completely separated in the “two vectors” setting (Fig. 4d).

### BIFC-dCas9/gRNA strategies for optimal chromosome imaging

Bimolecular fluorescence complementation (BIFC) was originally developed to validate protein-protein interactions through detection of fluorescence from the assembly of fluorescent protein fragments tethered to interacting proteins [25, 26]. BIFC measures the spatial and temporal changes for specific protein interactions but not for their noninteracting subunits. This property has been recently used to reduce the background fluorescence generated from free unbound fluorescent proteins, which could increase the signal-to-noise ratio and labeling efficiency for both RNA and protein labeling [27, 28].

**Fig. 5:**
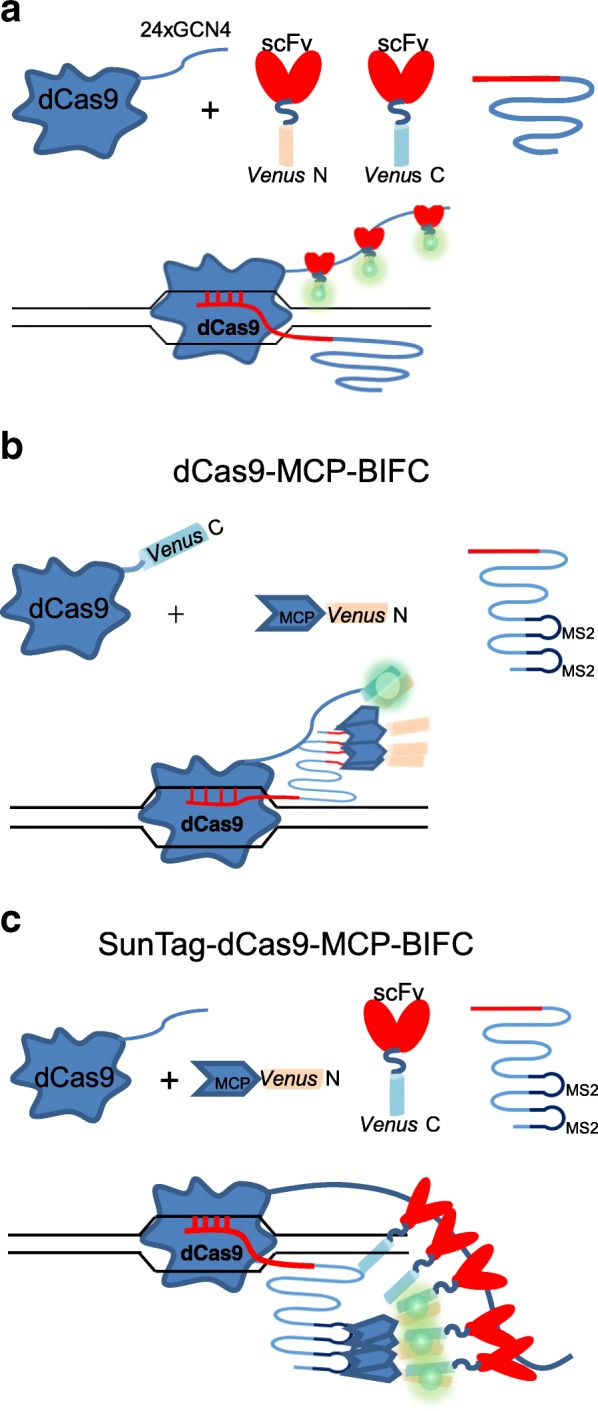
Schematic views of the different BIFC-dCas9/gRNA genome-labeling systems. **a** In scFv-BIFC, scFv-*Venus*N and scFv-*Venus*C are recruited to the SunTag of dCas9 where a functional *Venus* could be assembled. **b** In dCas9-MCP-BIFC, the *Venus*N is fused with MCP, which could be recruited to the dCas9/gRNA complex where it could interact with the *Venus*C fragment fused with dCas9 to form a functional *Venus* molecule. **c** In SunTag-dCas9-MCP-BIFC, the *Venus*C fragment is fused with scFv to be recruited to dCas9

**Fig. 6:**
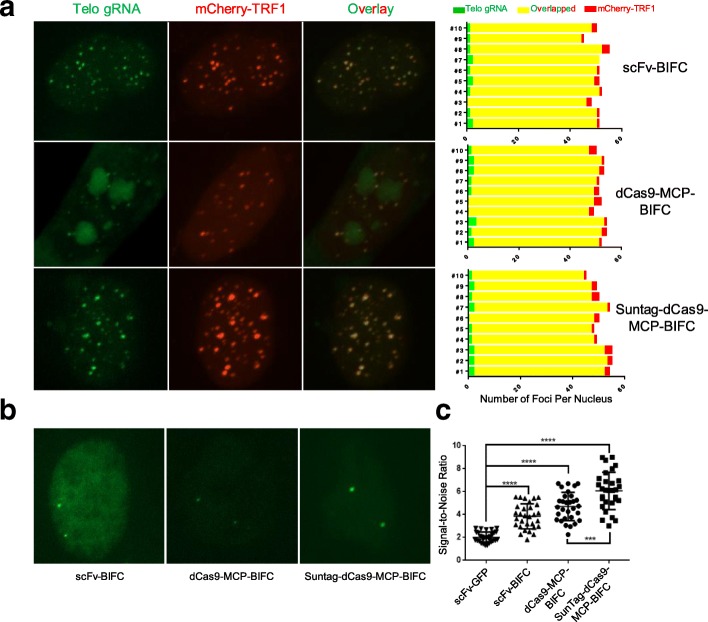
BIFC-dCas9/gRNA strategies showed high signal-to-noise ratio and no nonspecific foci. **a** Labeling of telomeres by different BIFC approaches. *Left panel*: the telomeres were labeled by *Venus* while the mCherry-TRF1 was used as control. *Right panel*: Quantification of telomere labeling specificity by co-localization with mCherry-TRF1 signals. **b** Comparison of different BIFC-dCas9/gRNA systems for labeling the endogenous human *MUC4* genomic locus. **c** Dot plots of signal-to-noise ratio in different BIFC-dCas9/gRNA genome-labeling systems in comparison with SunTag system. The same amounts of dCas9, gRNA, scFv-*Venus*, or scFv-*Venus*C/N expression constructs were transfected for different methods. The data are displayed as mean ± SD. Unpaired *t* test was used. ***p < 0.001, ****p< 0.0001

We designed several BIFC strategies to optimize CRISPR/dCas9 labeling (Fig. 5). First, split *Venus* N- and C-terminal parts (VN1–173 and VC155–238) were fused to the C-terminal of scFv to obtain scFv-VN and scFv-VC, respectively (Fig. 5a). They were co-transfected with SunTag-dCas9 and gRNAs for telomere and single genomic locus labeling. In dCas9-MCP-BIFC, VC155–238 were directly fused to the C-terminal of dCas9 while VN1–173 were fused to the C-terminal of MCP (MCP-VN) (Fig. 5b). Then they were co-transfected with gRNAs containing 3’ 2XMS2. In SunTag-dCas9-MCP-BIFC, scFv-VC was co-transfected with SunTag-dCas9, MCP-VN, and gRNA containing 3’ 2XMS2 (Fig. 5c). In scFv-BIFC, functional *Venus* molecules assembled from split *Venus* C- and N-terminal parts could be significantly enriched by the 24XGCN4 tag of SunTag-dCas9, whereas the fluorescence background from spontaneously assembled *Venus* proteins that is diffusely localized in the nucleus would be significantly reduced in comparison with scFv-*Venus*. In dCas9-MCP-BIFC and SunTag-dCas9-MCP-BIFC, functional *Venus* molecules could only be assembled within dCas9/gRNA complexes. These two strategies combine the specificity from both dCas9 and gRNA to increase labeling specificity. One could also speculate that more functional *Venus* molecules would be assembled in SunTag-dCas9-MCP-BIFC than in dCas9-MCP-BIFC, since the latter method could only form one functional *Venus* molecule per dCas9/gRNA complex. For both telomere (Fig. 6a) and *MUC4e* single genomic locus (Fig. 6b) labeling, those three BIFC methods showed similar labeling pattern and specificity to those of SunTag-dCas9 (Figs. 2 and 3), while there were absolutely no nonspecific foci in the –dCas9, –gRNA, and control gRNA samples (data not shown). More importantly, the signal-to-noise ratios of the MUC4e labeling by all three BIFC approaches greatly increased in comparison with the SunTag-dCas9 labeling systems when similar amounts of dCas9, gRNA, *Venus*, or split *Venus* expression constructs were transfected (Fig. 6c). In particular, SunTag-dCas9-MCP-BIFC showed the highest signal-to-noise ratio. The BIFC methods could also be used to label other genomic loci containing fewer repeats such as MUCI (40 repeats) and 197 M (48 repeats) foci (see Ref. [23], Additional file: Figure S6).

## Discussion

The recently developed CRISPR/Cas9 system provides a simple way to efficiently recognize and manipulate the targeted genome sequences in organisms [4–7]. In particular, several dCas9/gRNA genome-labeling methods that differentially tether fluorescent proteins with dCas9/gRNA complexes have been developed recently to track genome dynamics in living cells [11–20]. Here, we compared representative dCas9 and gRNA labeling strategies in the same experimental setting and cellular context. Our results have shown that, although they are able to label multiple foci at the same time, gRNA labeling strategies have intrinsic nonspecific labeling foci from the accumulation of gRNA transcripts, and this is more severe when more RNA-binding motifs are used. Finally, we developed several BIFC-dCas9/gRNA labeling methods that have a higher signal-to-noise ratio and also no nonspecific foci.

Several key issues have been mainly considered for optimizing the dCas9/gRNA genome labeling. First, careful validation of the observed fluorescent signals is necessary. For example, fluorescent in situ hybridization (FISH) has only been used in few studies [11], likely due to its technical difficulties, especially in combination with live cell imaging. Therefore, co-localization with other known labeling markers has been frequently used for telomere and centromere labeling [18–20]. In addition, co-labeling pairs of two nearby and distant foci could be performed [11, 14–16]. More essentially, adequate controls, especially negative controls, should be included to clarify potential nonspecific and specific artifacts. Moreover, simultaneously labeling several genome loci with different colors in the same cells is necessary to visualize dynamic interactions of genomic regions (e.g., the interaction between enhancer and promoter). Although direct labeling of different dCas9 orthologs with distinct fluorescent proteins has been developed [14, 15], this requires co-transfections of multiple dCas9 orthologs with their corresponding gRNAs into the same cell. Labeling gRNAs with different RNA motifs seems to be an easier way to image multiple genomic loci [16–20]. However, here we observed that the binding of corresponding proteins on such RNA motifs could lead to stabilization and accumulation of gRNA transcripts surrounding their transcription cassettes and formation of nonspecific labeling foci. Finally, the ultimate goal of dCas9/gRNA imaging is to label genomic loci with low- or nonrepetitive regions with a minimal number of targeted dCas9/gRNA complexes. Increasing the numbers of fluorescent proteins tethered to each dCas9/gRNA complex could lead to amplification of fluorescent signals. On the other hand, reducing the background noise, such as background fluorescence generated from free unbound fluorescent proteins, could also greatly enhance the signal-to-noise ratio. The BIFC-dCas9/gRNA methods developed here showed no nonspecific foci, especially those artifacts originating from gRNA transcripts. More importantly, the BIFC-dCas9/gRNA methods have a higher signal-to-noise ratio and could be the best choices for low-repeat-containing genome regions. The multiple-color choices for BIFC-dCas9/gRNA could be further extended to different bimolecular fluorescent complexes that have distinct spectrums [26] and could also be further combined with the CRISPRainbow system [17].

## Conclusions

We carefully compared current major CRISPR/dCas9/gRNA methodologies for genome labeling and provided the community with sets of validated reagents and protocols. In addition, we surprisingly discovered that in the gRNA-labeling strategies, accumulation of tagged gRNA transcripts could lead to significant nonspecific labeling foci in the absence and presence of dCas9. More importantly, we developed novel bimolecular fluorescence complementation (BIFC) methods that combine the advantages of current dCas9 labeling and gRNA labeling strategies. The BIFC-dCas9/gRNA methods demonstrate a higher signal-to-noise ratio compared to other existing dCas9/gRNA labeling systems and have absolutely no nonspecific foci.

## Methods

### Plasmids

The pHRdSV40-NLS-dCas9-24xGCN4_v4-NLS-P2A-BFP-dWPRE (Addgene #60910), pHR-scFv-GCN4-sfGFP-GB1-dWPRE (Addgene #60907), pHAGE-EFS-MCP-3XBFPnls (Addgene #75384), pHAGE-EFS-PCP-3XGFPnls (Addgene #75385), pLH-sgRNA1-2XMS2 (Addgene #75389), pAC1373-pX-sgRNA-25xPBSa (Addgene #71890), pAC1399-pX-sgRNA-20xPBSc (Addgene #71899), pAC1380-pX-sgRNA-5xPBSc (Addgene #71895), pAC1404-pCR8-mRuby2_NLSPUFa (Addgene #71902), pAC1403-pCR8-Clover_NLSPUFc (Addgene #71901), and pAC1358-pmax-NLSPUFc_VP64 (Addgene #71884) plasmids were obtained from Addgene, Cambridge, MA, USA. Their detailed information is listed in Ref. [23], Additional file 1: Table S1. The pcDNA3.1-dCas9 plasmid was described previously [24]. A list of the new plasmids generated in this work is provided in Ref. [23], Additional file 1: Table S2. Schemes and nucleotide sequences for those plasmids are also listed in Ref. [23]. A list of gRNAs used in this work is presented in Additional file 1: Table S3 (Ref. [23]).

#### BIFC plasmids

For scFv-*Venus*N 173 and scFv-*Venus*C 155 constructions, scFv-sfGFP-gb1-nls was digested with *Bam*HI and *Not*I. The gb1-nls was amplified from scFv-sfGFP-gb1-nls and then fused with *Venus*N 173/*Venus*C 155 (amino acid residues 1–173, 155–238, respectively) by polymerase chain reaction (PCR). The gb1-nls-*Venus*N 173/*Venus*C 155 fragments were inserted back to scFv-sfGFP-gb1-nls by In-Fusion cloning.

For NLS-HA-MCP *Venus*N 173 construction, pHAGE-EFS-MCP-3XBFPnls was digested by *Nco*I and *Xba*I. MCP and *Venus*N 173 (amino acid residues 1–173) were amplified by oligos containing nuclear localization signal (NLS)-hemaglutinin (HA) sequences and were fused by overlap PCR to generate an NLS-HA-MCP-*Venus*N173 fragment, which was then inserted into the digested pHAGE-EFS-MCP-3XBFPnls vector by Gibson ligation.

### Cell culture and transfection

HEK293T, B16, HeLa, and U2OS cells were cultured in Dulbecco’s modified Eagle’s medium (DMEM) supplemented with 100 unit/ml penicillin, 100 μg/ml streptomycin, and 10% fetal bovine serum. The cells were plated in 35-mm glass-bottom dishes the day before transfection, and were transfected with the indicated plasmids (see Ref. [23], Additional file 1: Table S4) by VigoFect (Vigorous Biotechnology, Beijing, China).

### Image acquisition and analysis

All images were taken on an UltraVIEW VoX spinning disc microscope (PerkinElmer, Hopkinton, MA, USA). The microscope incubation chamber was maintained at 37 °C and 5% CO_2_ when we acquired the images. Z-stack images were taken with a step size of 500 nm and enough steps to cover the depth of each nucleus.

Co-localization analysis was carried out using the Volocity software’s “Co-localization” function. The number counting of foci was performed by the “Measurement, Find Objects” function in Volocity software.

To measure the signal-to-noise ratio, a line was drawn across the spot first, and the “Plot Profile” function in ImageJ was used to generate an intensity profile [16, 19, 29]. To calculate the signal-to-noise ratio, the intensity profile was subjected to Gaussian calibration with a baseline, and then the highest intensity value of each peak was divided by the baseline value. The baseline (background noise) was defined as the fluorescence intensity of the nucleus (i.e., unbound fluorescent protein).

### ChIP analysis

The ChIP procedure was performed as described previously [24]. Briefly, cells were crosslinked with 1% formaldehyde for 5 min at room temperature, and the formaldehyde was then inactivated by the addition of 125 mM glycine for 5 min at room temperature. After cell lysis and sonication, the chromatin extracts were incubated with GFP-binding protein (GBP) beads overnight at 4 °C. After washing and reverse crosslinking, DNA was purified for qPCR quantification with specific primers (see Ref. [23], Additional file 1: Table S3).

### RNA extraction and RT-qPCR analysis

Cells were harvested 48 h post-transfection. Total cellular RNA was extracted using the Direct-zol™ RNA MiniPrep Kit (Zymo Research, Irvine, CA, USA). We used 100 ng total RNA to synthesize complementary DNA (cDNA) with the ImProm-II™ Reverse Transcriptase kit (Promega, Madison, WI, USA). The KAPA SYBR Universal 2× quantitative PCR kit (KAPA Biosystems, Wilmington, MA, USA) was used for qPCR reactions. Gene expression was normalized to the expression of the GAPDH gene. The qPCR primer sequences are included in Ref. [23], Additional file 1: Table S3.
